# Thoracic Temporal Subtraction Three Dimensional Computed Tomography (3D-CT): Screening for Vertebral Metastases of Primary Lung Cancers

**DOI:** 10.1371/journal.pone.0170309

**Published:** 2017-01-17

**Authors:** Shingo Iwano, Rintaro Ito, Hiroyasu Umakoshi, Takatoshi Karino, Tsutomu Inoue, Yuanzhong Li, Shinji Naganawa

**Affiliations:** 1 Department of Radiology, Nagoya University Graduate School of Medicine, Nagoya, Aichi, Japan; 2 Imaging Technology Center, Fujifilm Corporation, Tokyo, Japan; Universidad Francisco de Vitoria, SPAIN

## Abstract

**Purpose:**

We developed an original, computer-aided diagnosis (CAD) software that subtracts the initial thoracic vertebral three-dimensional computed tomography (3D-CT) image from the follow-up 3D-CT image. The aim of this study was to investigate the efficacy of this CAD software during screening for vertebral metastases on follow-up CT images of primary lung cancer patients.

**Materials and Methods:**

The interpretation experiment included 30 sets of follow-up CT scans in primary lung cancer patients and was performed by two readers (readers A and B), who each had 2.5 years’ experience reading CT images. In 395 vertebrae from C6 to L3, 46 vertebral metastases were identified as follows: osteolytic metastases (n = 17), osteoblastic metastases (n = 14), combined osteolytic and osteoblastic metastases (n = 6), and pathological fractures (n = 9). Thirty-six lesions were in the anterior component (vertebral body), and 10 lesions were in the posterior component (vertebral arch, transverse process, and spinous process). The area under the curve (AUC) by receiver operating characteristic (ROC) curve analysis and the sensitivity and specificity for detecting vertebral metastases were compared with and without CAD for each observer.

**Results:**

Reader A detected 47 abnormalities on CT images without CAD, and 33 of them were true-positive metastatic lesions. Using CAD, reader A detected 57 abnormalities, and 38 were true positives. The sensitivity increased from 0.717 to 0.826, and on ROC curve analysis, AUC with CAD was significantly higher than that without CAD (0.849 vs. 0.902, p = 0.021). Reader B detected 40 abnormalities on CT images without CAD, and 36 of them were true-positive metastatic lesions. Using CAD, reader B detected 44 abnormalities, and 39 were true positives. The sensitivity increased from 0.783 to 0.848, and AUC with CAD was nonsignificantly higher than that without CAD (0.889 vs. 0.910, p = 0.341). Both readers detected more osteolytic and osteoblastic metastases with CAD than without CAD.

**Conclusion:**

Our temporal 3D-CT subtraction CAD software easily detected vertebral metastases on the follow-up CT images of lung cancer patients regardless of the osteolytic or osteoblastic nature of the lesions.

## Introduction

Bone metastasis occurs at high rates in primary lung cancer. After or during primary lung cancer treatment, bone metastases occur in about 10–40% of patients [1–6]. The most common site of bone metastases in primary lung cancer patients is the spine, especially the thoracic vertebrae [3, 7]. Vertebral metastases can cause pain progression, compression fractures, and neural disturbances such as paralysis, which consequently compromise patients’ quality of life (QOL) [3, 6, 8]. Therefore, early detection and treatment to preserve QOL should be considered for these patients [1, 5, 6, 8]. Currently, the most helpful diagnostic imaging methods are spinal magnetic resonance imaging (MRI) and whole-body positron emission tomography (PET) [8, 9]. However, these diagnostic imaging methods are not routine in the follow-up of lung cancer patients who have none of the typical symptoms [5].

Chest computed tomography (CT) is routinely used to check for local recurrence and metastases in lung cancer patients [10, 11]. Although the detection rate of bone metastasis on CT is inferior to that on MRI and PET, incidental thoracic vertebral metastases are sometimes found on chest CT. Although the majority of lung cancer bone metastases are osteolytic in nature, osteoblastic and mixed metastases are sometimes observed [3]. On CT images, osteolytic metastases show lower attenuation than the surrounding bone, while osteoblastic ones show higher attenuation than the surrounding bone. However, the detection of vertebral metastases by computer-aided diagnosis (CAD) on CT is challenging [4, 12]. Temporal subtraction of chest radiograph is a CAD technique that initial digital image subtract from follow-up image on workstation, and it has already been commercialized [13, 14]. In addition, a temporal subtraction method of three-dimensional CT (3D-CT) has been reported recently [15–17]. Using temporal subtraction chest 3D-CT, both osteolytic and osteoblastic lesions derived from bone metastases of lung cancer can be detected. Thus, we have developed an original CAD software that can subtract the follow-up and initial 3D-CT of the spine with the application of non-rigid registration.

In the present study, we investigated whether temporal subtraction chest 3D-CT can contribute to the detection of vertebral metastases in lung cancer.

## Materials and Methods

Our retrospective study was approved by our institutional review board, and informed consent was waived (approval no. 636–3).

### Temporal Subtraction CAD of Chest CT

**Fig 1:**
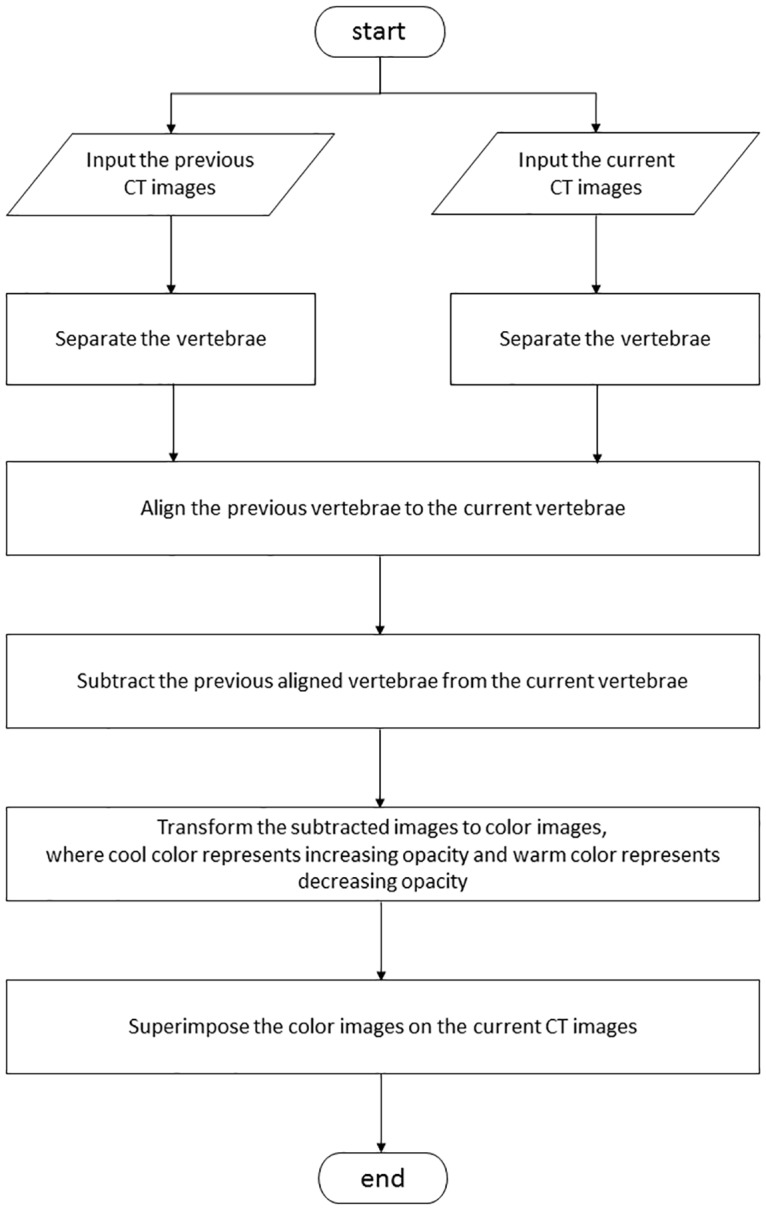
Flowchart of the temporal 3D-CT subtraction CAD.

**Fig 2:**
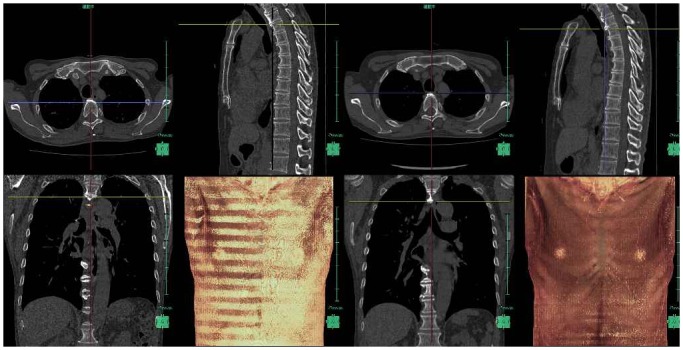
Original temporal 3D-CT subtraction CAD on the screen. The left half of the screen displays the follow-up CT images, and the right half of the screen displays the initial CT images.

Digital imaging and communications in medicine data of chest CTs were transferred into a workstation on which our original CAD software was installed. Figs 1 and 2 show the overall scheme of the subtraction 3D-CT procedure and examples of subtraction 3D-CT images, respectively. Sagittal and coronal multiplanar reconstruction (MPR) images from continuous thin-section CT images are automatically displayed on the workstation. Follow-up CT images are displayed on the left half and the initial images are displayed on the right half so that the radiologist can visually compare the follow-up and initial images on the monitor.

**Fig 3:**
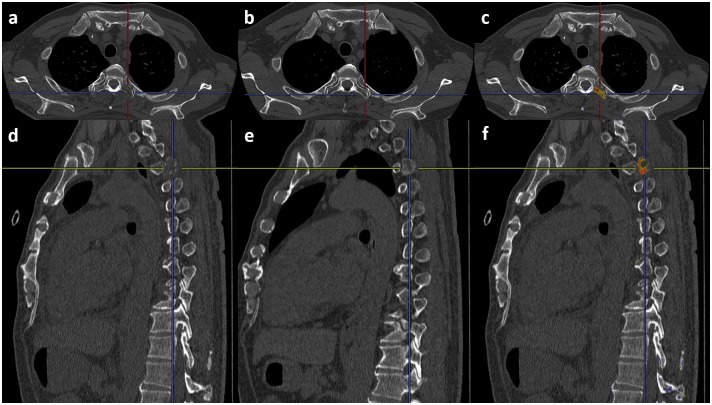
A case of osteolytic metastasis in the left transverse process of the third thoracic vertebra. (a) Follow-up axial image. (b) Initial axial image. (c) Result of the 3D-CT subtraction. The yellow and red colors indicate the metastasis. (d) Follow-up sagittal image. (e) Initial sagittal image. (f) Result of the 3D-CT subtraction. The yellow and red colors indicate the metastasis.

**Fig 4:**
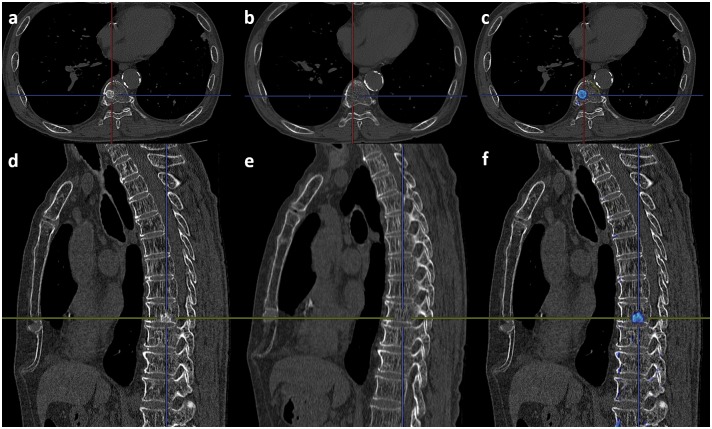
A case of osteoblastic metastasis in the body of the ninth thoracic vertebra. (a) Follow-up axial image. (b) Initial axial image. (c) Result of the 3D-CT subtraction. The blue color indicates the metastasis. (d) Follow-up sagittal image. (e) Initial sagittal image. (f) Result of the 3D-CT subtraction. The blue color indicates the metastasis.

First, the CAD software detected the centerline of the spine on both the follow-up and initial CT, and at this point, highly deformed vertebrae are detected as compression fractures. If there is a compression fracture, an alert is rendered on the monitor to caution against misregistration by 3D-CT subtraction. Next, corresponding vertebra between the follow-up and initial images is decided by 3D template matching along the centerline. After deciding on the right combination of vertebrae, 3D-CT subtraction using non-rigid deformation is performed. Finally, the colored difference of the 3D model is superimposed on the follow-up monochromatic MPR images. Osteolytic and osteoblastic lesions are indicated by cold (blue) and warm colors (yellow to red), respectively. Figs 3 and 4 show cases of vertebral metastases diagnosed by our CAD software.

### Reading Test Set

A set of 30 digital chest CT images for lung cancer follow-up was assembled randomly from the picture archiving and communication system (PACS) at our institution for the reading test. This sample size was determined assuming that the total reading time by a reader would be within 2 hours because long time reading causes reader’s fatigue.

Using the search function in the reading reports, chest CT reports, including all of those referring to “spine,” “bone metastasis,” and “lung cancer,” were extracted from the PACS. A chest radiologist with 21 years’ experience in reading chest CT images reviewed the extracted cases for the CT, MRI, and PET/CT findings and detailed clinical history, and selected 15 cases with new-onset bone metastases during the follow-up of lung cancer. The radiologist identified the lesion site (C6 to L3), and classified the lesions into the anterior component including the vertebral body or posterior component consisting of the vertebral arch, transverse process, and spinous process. A total of 46 vertebral metastases in 395 vertebrae (790 components) were identified as follows: osteolytic metastases (n = 17), osteoblastic metastases (n = 14), combined osteolytic and osteoblastic metastases (n = 6), and pathological fractures (n = 9). Thirty-six lesions were observed in the anterior component, and the remaining 10 lesions were observed in the posterior component. In addition, 15 cases without vertebral metastases were also extracted. Table 1 summarizes the 30 selected cases (24 male and 6 female; age range, 44–86 years). There were no significant differences in the male-to-female ratio, age, follow-up period, clinical stage based on TNM classification 7^th^ edition by union for international cancer control (UICC), and histology between the cases with and without metastasis.

**Table 1:** Comparison of the reading test cases between those with and without vertebral metastases.

		Metastases (–)	Metastases (+)	P value
**Female/male (n)**		2/13	4/11	0.651^#^
**Age (years)**	Median	68	61	0.093^##^
	Range	44–77	48–86	
**Follow-up period (months)**	Median	8	7	0.588^##^
	Range	1–49	1–14	
**Clinical stage: I / II / III / IV (n)**		8 / 4 / 1 / 2	3 / 2 / 5 / 5	0.075^###^
**Histology: AC / SCC / SCLC / Others (n)**		12 / 2 / 0 / 1	8 / 2 / 1 / 4	0.308^###^

^#^Fisher’s exact test;

^##^Mann–Whitney U test;

^###^Chi-square Test

AC, Adenocarcinoma; SCC, Squamous cell carcinoma; SCLC, Small cell carcinoma

All chest CT scans were performed with a 16-row or 64-row multidetector CT (Aquilion; Toshiba Medical, Tokyo, Japan) in the craniocaudal direction with inspiratory apnea between June 2006 and April 2014 at our hospital. The data were reconstructed with a slice thickness of 1.0 mm at 1.0-mm increments or a slice thickness of 0.5 mm at 0.5-mm increments.

### Image Reading Experiment

Two radiology residents, who each had 2.5 years’ experience in reading thoracic CT images, participated in the image reading experiment as readers A and B, respectively. Before the experiment, the readers were trained to use the CAD software using four cases that were not included in the test cases on the workstation. The two readers independently interpreted 30 cases of the follow-up and initial CT images on the workstation. The cases were presented in a random order. First, they were asked to detect vertebral bone metastases on the follow-up and initial chest CT sets without CAD [CAD (–)] and to record the confidence rating for the presence of the metastasis and the lesion site on the response sheet. Next, they were asked to detect vertebral bone metastases with CAD [CAD (+)] in the same 30 cases. A rating of 100 indicated that a vertebral metastasis was present with absolute certainty. Although no time limit was set, the total reading time for the 30 cases was measured.

### Statistical Analysis

The clinical background (gender, age, follow-up period, clinical stage, and histology) of the patients with and without vertebral metastases was compared by Fisher's exact test, the Mann–Whitney *U* test, and chi-square test. To calculate the sensitivity and specificity, the presence of bone metastases was assessed in 790 components. If metastases were found in both the anterior component and posterior component of a vertebra, both were judged as positive. Confidence ratings from 0 to 49 were considered negative and confidence ratings from 50 to 100 were considered positive. To examine the reproducibility between the two readers, the kappa statistic was calculated for both assessments with and without CAD. For each reader, the area under the curve (AUC) of the receiver operating characteristic (ROC) curve regarding the detectability of the vertebral metastases was compared between assessment with and without CAD by chi-square test.

Excel 2013 software (Microsoft Corp., Redmond, WA) and SPSS version 23.0 (IBM Corp., Armonk, NY) were used for statistical analysis. *P* <0.05 was considered statistically significant.

## Results

The total reading time of reader A was 1 h 41 min (3.4 min per case) and that of reader B was 1 h 50 min (3.7 min per case). The kappa statistic showed good reproducibility between the two readers for both assessments without CAD (0.672, p < 0.001) and with CAD (0.651, p < 0.001).

Table 2 shows the performance for the detection of vertebral metastases by each reader. Reader A detected 47 abnormalities on the CT images without CAD, and 33 of them were true-positive metastatic lesions. Using CAD, reader A detected 57 abnormalities, and 38 were true positives. The sensitivity was increased from 0.717 to 0.826, while the specificity was decreased from 0.981 to 0.974. On ROC curve analysis, the AUC with CAD was significantly larger than that without CAD (p = 0.021). Reader B detected 40 abnormalities on the CT images without CAD, and 36 of them were true-positive metastatic lesions. Using CAD, reader B detected 44 abnormalities, and 39 were true positives. The sensitivity was increased from 0.783 to 0.848, while the specificity was decreased from 0.995 to 0.993. On ROC curve analysis, the AUC with CAD was larger than that without CAD, although no significant difference was found (p = 0.341).

**Table 2:** Comparison of the detection of vertebral metastases with and without CAD.

	Reader A		Reader B	
	CAD (–)	CAD (+)	CAD (–)	CAD (+)
Number detected	47	57	40	44
Number of true positives	33	38	36	39
Number of false positives	14	19	4	5
Number of false negatives	13	8	10	7
Sensitivity	0.717	0.826	0.783	0.848
Specificity	0.981	0.974	0.995	0.993
AUC on ROC curve analysis	0.849*	0.902*	0.889	0.910

AUC, area under the curve; ROC, receiver operating characteristic; CAD, computer-aided diagnosis

*indicates a significant difference between CAD (**–**) and CAD (+) (p = 0.021)

Table 3 shows the performance of the two readers for the detection of vertebral metastases in the anterior and posterior components, separately. Using CAD, both readers detected more true-positive metastases and fewer false negatives in the posterior component than in the anterior component.

**Table 3:** Detection of anterior and posterior vertebral metastases with or without CAD.

	Reader A		Reader B	
	CAD (–)	CAD (+)	CAD (–)	CAD (+)
Anterior component				
Number detected	39	42	34	34
Number of true positives	30	31	32	32
Number of false positives	9	11	2	2
Number of false negatives	6	5	4	4
Posterior component				
Number detected	8	15	6	10
Number of true positives	4	7	4	7
Number of false positives	4	8	2	3
Number of false negatives	6	3	6	3

CAD, computer-aided diagnosis

Table 4 shows the detection performance for each type of bone metastasis. Reader A detected more osteolytic / osteoblastic metastases and pathologic fractures with CAD than without CAD, while Reader B detected more osteolytic and osteoblastic metastases with CAD than without CAD.

**Table 4:** Detection of each type of bone metastasis with or without CAD

		Reader A		Reader B	
Type of bone metastasis	Number	CAD(–)	CAD(+)	CAD(–)	CAD(+)
Osteolytic	17	12	14	12	14
Osteoblastic	14	9	11	11	12
Combined osteolytic and osteoblastic	6	6	6	6	6
Pathologic fracture	9	6	8	7	7

CAD, computer-aided diagnosis

## Discussion

The present study shows that our temporal 3D-CT subtraction CAD software can help radiologists to detect vertebral metastases, regardless of the osteolytic or osteoblastic nature of the lesions, during routine interpretation of a thoracic CT image for follow-up of primary lung cancer. Lung cancer regardless of the histopathological subtypes often occur bone metastasis. It causes skeletal related events defined as the need for radiotherapy or surgery, pathological fracture, spinal cord compression, and hypercalcemia [1, 3]. Vertebral metastases often result in pathological fracture or spinal cord compression, which can cause severe pain, paralysis, disturbance of gait, bladder and rectal disturbance. These complications cause significant morbidity and reduced QOL. Therefore, it is important for the physician and radiologist not to overlook them in routine check-up imaging even if a patient has no symptoms.

Bone scintigraphy has been used to detect bone metastasis; however, it has low detection rates for osteolytic lesions [18–21]. Although MRI is the gold standard non-invasive imaging method for the diagnosis of vertebral metastasis, it is not established as a method for lung imaging because of the long acquisition time required. Thus, bone scintigraphy and MRI are not recommended for routine check-ups in lung cancer patients without symptoms [10, 11]. Although ^18^F-FDG-PET/CT is another valuable imaging method for detecting bone metastasis [18, 20, 21], its high cost limits its use as a routine follow-up imaging method. For these reasons, only chest CT is currently performed as routine follow-up imaging in lung cancer patients without symptoms. However, chest CT images on lung and mediastinal window settings are not very suitable for demonstrating thoracic vertebral metastases, and lesions are sometimes overlooked by radiologists.

During interpretation of CT images, vertebral lesions are often estimated on the bone window setting, which has a window width of 1500 to 2000 HU [22–25]. On this setting, it is difficult for radiologists to visually detect the lesion on the monochromatic CT image because the difference in the opacity between normal bone tissue and lesion in both osteolytic and osteoblastic metastases is much smaller than the window width. Thus, we designed our CAD software to emphasize the results of bone subtraction by adding color to the monochromatic CT images. The color image could also be used to distinguish between osteolytic lesions (displayed in red and yellow) and osteoblastic lesions (displayed in blue). Because osteolytic vertebral metastases cause pathological fractures more often than osteoblastic ones, differential diagnosis between them is important.

In the reading experiment, detection of metastases by both readers improved more in the posterior component using CAD than without CAD. This result might have been caused by the difference in the 3D form between the vertebral body as anterior components and posterior components. On sagittal view of the 3D-CT, the vertebral body usually looks like a square, whereas the posterior component shows a much more complicated shape. Therefore, it was difficult for the readers to detect metastases in the posterior component without CAD. CAD improved the visibility of the bone lesion image in this complicated structure by adding color. It is important to identify bone metastases in the posterior component in lung cancer patients because they can cause spinal cord compression as well as vertebral body metastasis.

Although our reading test cases included only primary lung cancer patients in the present study, our 3D-CT subtraction CAD software could be applied in the detection of vertebral metastases not only in lung cancer but also other malignant tumors because the readers could detect more of both osteoblastic and osteolytic metastases when evaluating the images with CAD than without CAD. Bone metastases are often found in patients with breast and prostate cancer. Most patients with breast cancer have predominantly osteolytic metastases, although 15 to 20% of them have predominantly osteoblastic lesions, while patients with prostate cancer have predominantly osteoblastic metastases [26, 27]. In these tumors, vertebral metastases are found incidentally on chest CT when checking for pulmonary or thoracic lymph node metastasis. CAD may provide an easy way to follow up patients with a history of malignancy on regular CT.

This initial study indicated an improvement of the 3D-CT subtraction CAD. It was automatic anatomical labeling function of the thoracolumbar spine. In the reading test, the two readers took about 3 min per patient with CAD, and most of the time was spent evaluating the site of metastasis not detecting the metastasis itself. Accurately locating the lesion site is an important task for radiologists. However, it is also challenging to create computer software that can anatomically label vertebrae accurately and automatically [28]. Recently, Scholtz et al. created software that achieved correct automatic labeling in 72 of 77 patients (93.5%) [25]. A combination of vertebral subtraction and automatic labeling may save time for radiologists. Since thin-section CT images reconstructed from the routine chest CT scan are used for this temporal 3D-CT subtraction CAD, it is not necessary to add special CT device. Therefore, the cost of the examination will not change.

This study has several limitations. First, it was a retrospective and single-center study. All cases in the reading test were recruited randomly from a lung cancer database on our PACS. Thus, our study is limited by selection bias. Next, the diagnosis of vertebral metastasis was based on clinical and radiological findings, not on the histological examination. Although invasive vertebral biopsy is the gold standard for the demonstration of bone tumors [6, 19], in the clinical setting, treatment for vertebral metastasis does not always require pretreatment pathological confirmation because vertebral metastases grow rapidly and can lead to neurological symptoms. Third, our study is lack of validation dataset.

In conclusion, our temporal 3D-CT subtraction CAD software easily detected vertebral metastases on the follow-up CT images of lung cancer patients regardless of the osteolytic or osteoblastic nature of the lesions. This software could prevent radiologists from overlooking asymptomatic vertebral metastases during routine interpretation of thoracic CT images for follow-up of lung cancer.

## Supporting Information

S1 Table Characteristics of reading test cases. (DOCX)
