# Supplementary material for: Thoracic Temporal Subtraction Three Dimensional Computed Tomography (3D-CT): Screening for Vertebral Metastases of Primary Lung Cancers
Source: PLoS One. 2017 Jan 17;12(1):e0170309. doi: 10.1371/journal.pone.0170309 (PMC5240957; doi:10.1371/journal.pone.0170309)
Supplement: S1 Table — (DOCX) [file pone.0170309.s001.docx]

**S1 Table** Characteristics of reading test cases

| Case No. | Vertebral Metastasis | Gender | Age (years) | Follow-up period (months) | CT scan range | Histology | Clinical stage |
| --- | --- | --- | --- | --- | --- | --- | --- |
| 1 | (+) | Female | 54 | 7 | Th1-L2 | AC | IV |
| 2 | (+) | Female | 55 | 9 | C6-L1 | AC | II |
| 3 | (+) | Female | 56 | 1 | Th2-Th12 | AC | I |
| 4 | (+) | Female | 76 | 14 | Th2-L3 | AC | I |
| 5 | (+) | Male | 48 | 3 | C7-L1 | Undifferentiated carcinoma | III |
| 6 | (+) | Male | 53 | 1 | Th1-Th12 | SCC | II |
| 7 | (+) | Male | 54 | 9 | Th2-L1 | SCLC | IV |
| 8 | (+) | Male | 59 | 8 | Th1-TH12 | AC | IV |
| 9 | (+) | Male | 61 | 9 | C7-L2 | AC | III |
| 10 | (+) | Male | 62 | 4 | C7-L1 | AC | IV |
| 11 | (+) | Male | 66 | 4 | Th2-L3 | Adenosquamous carcinoma | I |
| 12 | (+) | Male | 66 | 12 | C6-L2 | Pleomophic carcinoma | III |
| 13 | (+) | Male | 67 | 5 | Th2-TH11 | AC | IV |
| 14 | (+) | Male | 72 | 5 | Th1-TH12 | SCC | III |
| 15 | (+) | Male | 86 | 9 | C7-L1 | NSCLC | III |
| 16 | (-) | Female | 46 | 1 | Th1-L2 | AC | I |
| 17 | (-) | Female | 67 | 49 | C7-L2 | AC | 1 |
| 18 | (-) | Male | 44 | 3 | Th2-Th12 | AC | III |
| 19 | (-) | Male | 56 | 9 | C7-L2 | AC | I |
| 20 | (-) | Male | 62 | 1 | Th1-L1 | NSCLC | II |
| 21 | (-) | Male | 68 | 2 | Th1-L1 | AC | I |
| 22 | (-) | Male | 68 | 13 | Th1-L2 | AC | I |
| 23 | (-) | Male | 68 | 19 | C7-L2 | AC | II |
| 24 | (-) | Male | 70 | 2 | Th2-Th12 | AC | II |
| 25 | (-) | Male | 70 | 8 | Th1-Th12 | SCC | IV |
| 26 | (-) | Male | 74 | 46 | Th1-L2 | AC | I |
| 27 | (-) | Male | 75 | 13 | Th2-Th12 | SCC | I |
| 28 | (-) | Male | 76 | 28 | Th1-L1 | AC | II |
| 29 | (-) | Male | 77 | 1 | Th2-L1 | AC | I |
| 30 | (-) | Male | 77 | 8 | C7-Th12 | AC | IV |

AC, adenocarcinoma; SCC, squamous cell carcinoma, NSCLC, non-small cell carcinoma;

SCLC, small cell carcinoma
